# ECRG2/SPINK7 Tumor Suppressor as Modulator of DNA Damage Response

**DOI:** 10.3390/ijms25115854

**Published:** 2024-05-28

**Authors:** Harsh Patel, M. Saeed Sheikh, Ying Huang

**Affiliations:** Department of Pharmacology, State University of New York Upstate Medical University, 750 East Adams Street, Syracuse, NY 13210, USA; harshpatel.niper@gmail.com

**Keywords:** tumor suppressor, ECRG2, p53, DNA damage response, cell death

## Abstract

Esophageal Cancer-Related Gene 2 (*ECRG2*), also known as Serine Peptidase Inhibitor Kazal type 7 (*SPINK7*), is a novel tumor suppressor gene from the *SPINK* family of genes that exhibits anticancer potential. *ECRG2* was originally identified during efforts to discover genes involved in esophageal tumorigenesis. *ECRG2* was one of those genes whose expression was absent or reduced in primary human esophageal cancers. Additionally, absent or reduced *ECRG2* expression was also noted in several other types of human malignancies. *ECRG2* missense mutations were identified in various primary human cancers. It was reported that a cancer-derived ECRG2 mutant (valine to glutamic acid at position 30) failed to induce cell death and caspase activation triggered by DNA-damaging anticancer drugs. Furthermore, *ECRG2* suppressed cancer cell proliferation in cultured cells and grafted tumors in animals and inhibited cancer cell migration/invasion and metastasis. ECRG2 also was identified as a negative regulator of Hu-antigen R (HuR), an oncogenic RNA-binding protein that is known to regulate mRNA stability and the expression of transcripts corresponding to many cancer-related genes. *ECRG2* function is important also for the regulation of inflammatory responses and the maintenance of epithelial barrier integrity in the esophagus. More recently, *ECRG2* was discovered as one of the newest members of the pro-apoptotic transcriptional targets of p53. Two p53-binding sites (BS-1 and BS-2) were found within the proximal region of the *ECRG2* gene promoter; the treatment of DNA-damaging agents in cancer cells significantly increased p53 binding to the *ECRG2* promoter and triggered a strong *ECRG2* promoter induction following DNA damage. Further, the genetic depletion of *ECRG2* expression significantly impeded apoptotic cell death induced by DNA damage and wild-type p53 in cancer cells. These findings suggest that the loss of *ECRG2* expression, commonly observed in human cancers, could play important roles in conferring anticancer drug resistance in human cancers. Thus, ECRG2 is a novel regulator in DNA damage-induced cell death that may also be a potential target for anticancer therapeutics.

## 1. Introduction

Esophageal Cancer-Related Gene 2 (*ECRG2*), also known as Serine Peptidase Inhibitor Kazal type 7 (*SPINK7*), is a putative tumor suppressor gene that was originally discovered by studies attempting to identify genes that were involved in human esophageal cancer [1]. Subsequent studies have identified numerous biological functions linked to *ECRG2* including growth inhibition [1,2], the induction of apoptosis [3,4], the suppression of epithelial–mesenchymal transition (EMT) and metastasis [5], the maintenance of cellular ploidy [6] and epithelial barrier integrity, the regulation of inflammation [7], increasing proteosome degradation of the RNA-binding protein HuR [3], and the modulation of DNA damage-induced responses [4].

In addition, *ECRG2* somatic missense mutations were reported in different human malignancies. Cancer-derived ECRG2 mutations appear to alter its function. For example, the V30E mutant identified in human lung cancer failed to inhibit tumor cell growth, significantly abolished DNA damage-induced cell death, and was linked to the acquisition of anticancer drug resistance [3,4]. Further, genomic variations in the *ECRG2* gene promoter or polymorphisms in its sequence corresponding to the 3′ untranslated region (UTR) were found to affect the regulation and expression of *ECRG2* [4,8]. Thus, multiple lines of evidence indicate that ECRG2 appears to be an important tumor suppressor that warrants more attention and investigation. In this review, we will discuss various biological functions of ECRG2 in relation to cancer biology, DNA damage response, and therapeutics.

## 2. Identification and Molecular Characteristics of *ECRG2*

*ECRG2* was originally identified by Su et al. [9] aiming to discover esophageal cancer-related gene(s) in cancer patients in Linxian, a county in northern China that has the highest incidence and mortality rate of esophageal cancer (EC) in the world. Using the RT-PCR differentiate display approach, Su et al. [9] identified eighteen mRNA fragments that were differentially expressed in the EC tissues versus those in the normal esophageal epithelia [9]. Among them, 13 mRNA fragments were found to be only expressed in the normal esophageal epithelia but not in esophageal cancer (EC), whereas 5 mRNA fragments were only detected in the EC but not in the normal esophageal epithelial [9]. The mRNA fragment that was later known to be the transcript of *ECRG2* was among those transcripts that were only detected in normal esophageal tissues but not in cancerous tissues [9]. This finding was later confirmed by other studies [10,11]. Thus, these initial studies suggest that *ECRG2* might have an important role in the development of esophageal cancer in humans.

**Figure 1:**
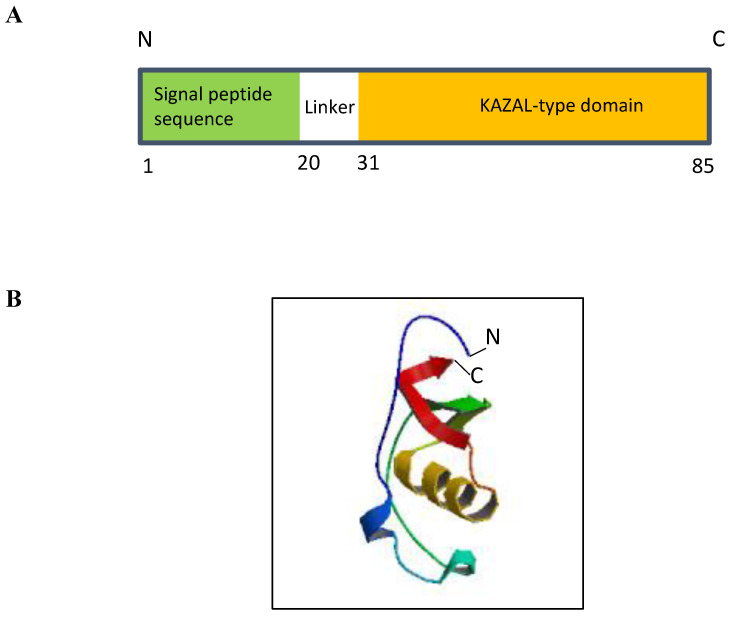
Structure of ECRG2 protein. (**A**) ECRG2 is predicted to contain an N-terminal signal peptide from amino acids 1–20 and a conserved KAZAL-type domain at its C-terminal from amino acids 31–85. (**B**) ModeBase predicted structure of 20–85 ECRG2 showing two alpha helices and three beta sheets [18].

The genomic location of *ECRG2* was mapped to human chromosome 5q32 [10]. This chromosomal region is frequently perturbed by genetic aberrations and allelic loss in various human cancers, including esophageal tumors [12,13]. *ECRG2* consists of four exons and three introns that are spread across a ~3.5 kilobase at chromosome 5q [10]. ECRG2 is a small protein composed of 85 amino acids with a predicted molecular mass of 9.23 kDa (Figure 1A) [10]. ECRG2 protein harbors an N-terminal signal peptide (a.a. 1–20), a central linker region, and a C-terminal conserved Kazal-type serine peptidase inhibitor domain (a.a. 31–85) (Figure 1A) that is shared by all serine protease inhibitor Kazal (SPINK) family proteins (discussed below) [10,14]. Structurally, ECRG2 is composed of two alpha helices and three beta sheets (Figure 1B) [14]; the Kazal-type domain of ECRG2 contains six conserved cysteine residues (Cys32, Cys45, Cys53, Cys64, Cys67, and Cys85) (Figure 1B), which form three intra-molecular disulfide bonds (Cys32–Cys67, Cys45–Cys64, and Cys53–Cys85) [10,14]. Studies have demonstrated that the correct formation of these disulfide bonds is important for protein structure and function [14,15]. ECRG2 protein has an N-terminal signal peptide sequence and is predicted to be a secreted protein (Figure 1A). Experimental evidence has demonstrated that ECRG2 is indeed a secretory protein [10]. In addition, ECRG2 is also widely distributed in the cytosol; it co-localized with microtubules during the interphase and mitotic phase of cell cycles [10,15] and localized at the centrosome during the G_1_/S phase and kinetochore during mitosis [6]. ECRG2 disruption has been reported to result in centrosome amplification and spindle checkpoint defects [6]. Thus, evidence suggests that ECRG2 is a multi-functional protein that plays important roles in the regulation of microtubule dynamics and cell cycle progression [10,15] as well as chromosome stability [6]. Further, recent studies have also identified a shorter isoform of ECRG2. This shorter ECRG2 isoform comprises only 59 amino acids and lacks the first 29 amino acids compared to the full-length ECRG2 protein [16,17]. The amino acid sequence of the shorter ECRG2 isoform shares 95% similarity to the C-terminus of the full-length ECRG2 [16,17]. Currently, there is a paucity of information about its expression profile and function.

## 3. SPINK Family Proteins and SPINK7/ECRG2 in Human Cancers

Due to the presence of a Kazal-type domain, ECRG2 is also termed as Serine Peptidase Inhibitor Kazal type 7 (SPINK7) and grouped with other SPINK family proteins, which are characterized by the presence of at least one Kazal-type domain in their structures [1]. The Kazal domain (40–60 amino acids) is evolutionarily conserved among different species [16] and is composed of one α helix and a three-stranded anti-parallel β-sheet with six cysteine residues forming three intra-domain disulfide bridges (Figure 1B) [19]. To date, ten members of *SPINK* family have been identified including *SPINK1*, *SPINK2*, *SPINK4*, *SPINK5*, *SPINK6*, *SPINK7/ECRG2*, *SPINK8*, *SPINK9*, *SPINK13*, and *SPINK14* [20]. Interestingly, seven of the ten *SPINK* genes identified are clustered at a region of chromosome 5q32; the *ECRG2/SPINK7* gene resides within this gene cluster along with six other *SPINK* genes (*SPINK1*, *SPINK5*, *SPINK6*, *SPINK9*, *SPINK13*, and *SPINK14*) [21].

SPINK proteins are expressed in various tissues, where they regulate serine peptidases and proteolysis activities [22,23,24,25]. However, multiple studies have shown that the expression of *SPINK* genes is dysregulated in human cancers. For example, *SPINK1* overexpression was detected in cancers of the gastrointestinal tract, lung, kidney, bladder, prostate, ovary, breast, and testis [26]. Moreover, the elevated expression of *SPINK1* was shown to enhance the growth, migration, and invasion of hepatocellular carcinoma cells (HCC) and was associated with poor prognosis in HCC patients [27]. SPINK6 was shown to promote metastasis of nasopharyngeal carcinoma by the activation of epithelial growth factor receptors [28]. Conversely, some SPINK family members were found to have anti-proliferative activity. The loss of *SPINK4* expression was detected in colorectal cancer (CRC) and lower *SPINK4* expression was linked with reduced disease-free survival in CRC patients [29]. Sun et al. [30] showed that *SPINK5*, which is an important biomarker of oral squamous cell carcinoma (OSCC), can prevent the development of OSCC by the inhibition of the Wnt/β-catenin signaling pathway. In addition, a reduction of *ECRG2/SPINK7* was also found in several other human malignancies, i.e., head and neck squamous cell cancer, cervical squamous cell carcinoma, and endocervical adenocarcinoma, which was associated with reduced disease-free survival in patients [4]. Although further studies are warranted to elucidate the exact molecular function of SPINK proteins in human malignancies, the available line of evidence nonetheless suggests that *SPINK* family genes play crucial roles in cancer development in humans.

## 4. Biological Activities of ECRG2/SPINK7

### 4.1. Inhibition of Cancer Cell Metastasis

Like other SPINK family proteins, ECRG2/SPINK7 was shown to have serine peptidase inhibitory activity. ECRG2 protein was demonstrated to inhibit the activity of a serine protease known as urokinase-type plasminogen activator (uPA), an enzyme involved in the conversion of inactive plasminogen into active plasmin, which is important in cancer metastasis [5,10]. Evidence showed that secreted ECRG2 directly binds to uPA and its receptor uPAR present on the cell surface to form a complex [5,31]. It was proposed that this complex disrupts the uPA pathway by three different mechanisms: (1) the inhibition of uPA/plasmin and matrix metallopeptidase 2 (MMP2)-mediated proteolytic activity [5], (2) the prevention of uPAR interaction with α3β1 and α5β1 integrin followed by the inhibition of integrin-mediated activation of the Src/ERK pathway [31], and (3) the prevention of uPA-mediated cleavage of uPAR, which leads to the inhibition of uPAR interaction with and activation of a G protein-coupled receptor, FPRL1 [32]. It was shown that the inhibitory action of ECRG2 on serine protease uPA suppresses the degradation of the extracellular matrix (ECM) and cancer cell invasion and metastasis [5,31,32].

### 4.2. Regulation of Inflammatory Responses

The protease inhibitor activity of ECRG2/SPINK7 protein has also been linked to an allergic condition of the esophagus in humans known as eosinophilic esophagitis (EoE). Recent studies found that endogenous *ECRG2/SPINK7* was depleted in esophageal tissue biopsies from EoE patients [7,33]. Further studies demonstrated that the loss of *ECRG2/SPINK7* expression led to the activation of esophageal eosinophils through elevated uPA/uPAR activity [7]. Moreover, the silencing of *ECRG2/SPINK7* expression disrupted epithelial barrier integrity and induced the release of pro-inflammatory mediators such as thymic stromal lymphopoietin, IL-1β, and TNF-α [7]. Hence, it is evident that *ECRG2/SPINK7* plays an important role in protecting esophageal barrier function and keeping epithelial inflammatory factors in check [7]. It is well-recognized that chronic inflammation is an important risk factor in esophageal cancer development [34,35]. The findings by Azouz et al. [7] thus suggest that esophagus chronic inflammation developed due to the loss of *ECRG2/SPINK7* expression may be a considerable risk factor for esophageal cancer formation.

Recently, Zhao et al. [36] showed that ECRG2/SPINK7 played an important protective role in chemically induced colitis in animals and that ECRG2/SPINK7-deficient animals were highly susceptible to induced colitis. They found that ECRG2/SPINK7 was significantly elevated in dextran sodium sulfate (DDS)-induced colitis in mice and, interestingly, cells with elevated ECRG2/SPINK7 expression in colitis tissues were mainly the neutrophils [36]. Moreover, ECRG2/SPINK7-deficient mice (SPINK7^−/−^) developed more severe colitis with more extensive ulcers, a higher disease activity index, and more severe body weight loss compared with their wild-type littermates; the loss of ECRG2/SPINK7 also impaired the recovery of colitis after DDS exposure was stopped [36]. The expression of chemokines/cytokines including CXCL1, CXCL2, CCL2, CCL3, CCL4, IL-1β, IL-6, CCL11, and CCL17 was also much higher in the SPINK7^−/−^ colitis tissues than that in SPINK7^+/+^ tissues [36]; elevated expression of inflammatory cytokines is expected to lead to more severe inflammation in colonic tissues. These results could suggest that the presence of or elevated ECRG2/SPINK7 expression is important for the protection/reduction of inflammation and damage to colonic tissues exposed to colitis-inducing chemicals. These findings are rather interesting; however, the molecular mechanisms involving SPINK7 modulation of colonic inflammation and cytokine/chemokine productions are currently not clear and need to be further investigated.

### 4.3. Roles in the Maintenance of Genome Integrity and Cancer Cell Suppression

ECRG2 is also implicated in proper centrosome duplication during the interphase and orderly chromosome segregation during mitosis [6]. Cheng et al. [6] showed that ECRG2 is crucial for the localization of p53 to centrosomes, and the silencing of *ECRG2* expression abolished p53 localization to centrosomes. Previous studies have shown that p53 mitotic centrosome localization is crucial in keeping genome integrity [37]. Further, Cheng et al. [6] also showed that *ECRG2* knockdown in cells led to the increased ubiquitination and degradation of p53, reduced p21 (a p53 target) at the protein level, and the increased activity of cyclin E/CDK2, which ultimately caused centrosome amplification. Decreased ECRG2 protein levels also impaired spindle assembly checkpoints by reducing BUBR1 (budding uninhibited by benzimidazoles-related 1) protein levels [6]. Thus, decreased *ECRG2* expression ultimately leads to chromosomal instability and aneuploidy [6], the characteristics of premalignant lesions and cancer [38,39]. ECRG2 has also been shown to affect the growth of cancer cells. An initial study by Cui et al. [1] showed that exogenously expressed ECRG2 inhibited esophageal cancer cell proliferation and induced cell death. They showed that ECRG2 directly interacted with metallothionein 2A (MT2A), and the ECRG2-mediated modulation of MT2A function was thought to be a possible mechanism via which ECRG2 suppresses esophageal cancer cell growth [1]. Although the gene was initially identified from esophageal tissues, the growth inhibitory effect of ECRG2 is not limited to esophageal cells. Studies have also shown that the overexpression of ECRG2 also induced apoptosis in cancer cells derived from different tissues including the colon, breast, lung, liver, and cervix [2,3,4]. The induction of cell death by ECRG2 was shown to be associated with the activation of caspases 8, 9, and 3 and cleavage of PARP in lung, breast, and cervical cancer cells [3,4] or with the modulation of nuclear factor-κB, matrix metalloproteinase 2, and E-cadherin in hepatic cancer cells [2]. Furthermore, a recent study by Lucchesi et al. [3] has shown that ECRG2 mediates its apoptotic effect by the negative regulation of Hu-antigen R (HuR, also known as ELAV1) and the X chromosome-linked inhibitor of apoptosis protein (XIAP). HuR is known to be a key RNA regulatory protein that affects the stability of numerous target mRNAs [40], while XIAP is an apoptosis inhibitor that inhibits the activation of caspases 3, 7, and 9 [41]. Lucchesi et al. [3] showed that the overexpression of ECRG2 caused a significant reduction in XIAP mRNA levels, which was not associated with the inhibition of the XIAP promoter but rather with alterations in XIAP mRNA stability, which is known to be stabilized by HuR [42]. Lucchesi et al. [3] further showed that ECRG2 promoted the proteasomal degradation of HuR and thus modulated XIAP mRNA levels by suppressing its mRNA stabilizer HuR [3]. It is of note that HuR also regulates many targeted mRNAs encoding proteins that are important in the regulation of cell cycle, proliferation, cell survival, and apoptosis (reviewed in [43]). Thus, ECRG2-mediated proteasomal degradation of HuR protein may have even broader effects on cellular functions in general and apoptosis in particular.

Interestingly, while exogenously expressed ECRG2 induced cell death in cancer cells, it did not appear to affect the growth of non-cancerous breast epithelial cells [3]. This suggests that ECRG2-mediated cell growth control exhibits cancer-specific selectivity. In this context, studies by Song et al. [2] demonstrated that adenovirus-mediated ECRG2 expression suppressed hepatic cancer cells grown on nude mice, with no apparent toxicity in the animals. These studies together demonstrated that ECRG2 may have anticancer therapeutic potential. Future studies are certainly needed to further investigate this issue.

### 4.4. ECRG2 Is an Important p53 Target and Effector in DNA Damage Response

Tumor suppressor p53 is known to play a key role in DNA damage response [44,45]. Following DNA damage, p53 is activated via post-translational modifications such as phosphorylation and acetylation, which lead to the stabilization and accumulation of p53 protein in the nucleus [44]. The p53 protein molecules accumulated inside the nucleus form a tetramer, which binds to the response elements located within the promoter or intronic regions of the target genes to activate their transcriptions [46]. These target genes are involved in an array of biological processes such as cell cycle arrest (e.g., Cyclin-Dependent Kinase Inhibitor 1A (*CDKN1A)/p21*, *GADD45a*, *14–3-3σ*), autophagy (e.g., *DRAM*), and apoptosis (e.g., *BAX*, *PUMA*, *DR5*) [46,47]. In the past two decades, multiple pro-apoptotic downstream targets of p53 have been characterized [48]; however, the inactivation of none of these genes was able to phenocopy the deficiency in apoptotic signaling observed in p53-null cells [44]. It was proposed that successful tumor suppression by p53 may require the functional redundancy of multiple downstream target genes with pro-apoptotic activity [49]. Alternatively, it was suggested that distinct pro-apoptotic target genes of p53 are induced by stress stimuli and in a cell-type-specific manner [50]. This conjecture paved the path for the continued discovery and characterization of novel pro-apoptotic targets of p53.

A recent study by Patel et al. [4] has demonstrated that *ECRG2* is a novel p53 target gene and an integral part of p53-mediated DNA damage response. ECRG2 mRNA and protein were significantly induced by the treatments of anti-neoplastic DNA-damaging agents such as etoposide (topoisomerase II inhibitor) or melphalan (an alkylating agent) [4]. Patel et al. [4] found that DNA damage-induced *ECRG2* expression was associated with the activation of the *ECRG2* promoter. Further analyzing the *ECRG2* promoter, they discovered that the region of the *ECRG2* promoter (from −1000 bp to transcription starting site) harbored regulatory binding sites for p53, p63, and OCT-1, the transcription factors important for the regulation of DNA damage responses [4]. Two putative p53-binding sites were identified within the *ECRG2* gene promoter, one (p53-BS-1) residing at −844 to −825 and another (p53-BS-2) localizing at −587 to −568. Following DNA damage, p53 was substantially recruited to the p53-binding sites within the *ECRG2* promoter, which resulted in the significant induction of *ECRG2* promoter activity and mRNA expression [4]. They further showed that etoposide-induced *ECRG2* promoter activation occurred only in the RKO p53^+/+^ cells, but not in RKO p53^−/−^ cells. More importantly, the disruption of *ECRG2* by gene targeting significantly diminished etoposide-induced apoptosis in cells even with the strong induction of wild-type p53 [4]. Such results suggest that the absence of ECRG2 blunts p53-mediated apoptosis following DNA damage and, like other p53 target proteins such as PUMA, NOXA, and DR5, ECRG2 acts as an effector of p53 to modulate DNA damage-induced cell death. It is of note that reduced or absent *ECRG2* expression was found in significant portions of human cancers [4]. It is possible that the insufficient function of ECRG2 may play an important role in anticancer drug resistance in human cancer.

## 5. ECRG2/SPINK7 Dysregulation, Mutations, and Other Genomic Variants

p53 is often inactivated by deletion and mutation in human cancers [51,52]. Since *ECRG2* is shown to be a transcriptional target of p53, one possible mechanism of loss of *ECRG2* mRNA expression in human cancers may be due to the inactivation of p53 function by deletion or mutations. Recently, Patel et al. [4] demonstrated that the expression of tumor-derived mutant p53-R273H caused a decrease in ECRG2 protein expression in RKO p53^−/−^ colon cancer cells, which was previously shown to compromise the transcriptional activation function of wild-type p53 [53,54]. A recent study showed that while ECRG2/SPINK7 was downregulated in less aggressive oral squamous cell carcinoma (OSCC), the protein levels of p53 remained elevated [55]. Given that p53 missense mutations such as p53-R273H are frequent in clinical cases of OSCC, which targets the DNA binding domain of wild-type p53 [56] and compromises the transcriptional activation function [53,54], it is likely that mutations in the upstream transcription activator p53 may lead to decreased levels of ECRG2/SPINK7 protein in clinical cases of human cancers. Further in-depth studies are needed to dissect the link between p53 status and *ECRG2* expression in normal and cancer tissues in humans.

Multiple studies have found a significant correlation between a short tandem repeat (STR) polymorphism (TCA3/TCA3) in the 3′-untranslated region (UTR) of *ECRG2* and increased incidence as well as poor prognosis of esophageal [8,57,58,59] and oral [60] cancers in various patient populations. Zhang et al. [8] showed that microRNA 1322 (miR-1322), which was found overexpressed in esophageal carcinoma, preferentially bound to TCA3 allele present at the site of STR polymorphism within *ECRG2* 3′-UTR and downregulated *ECRG2* expression. Due to its robust downregulation in gastric cancer, salivary extracellular RNA (exRNA) of *ECRG2* was utilized for the configuration of a biomarker panel for the noninvasive detection of gastric cancer [61]. Conversely, *ECRG2* expression was significantly upregulated in human chromophobe renal cell carcinoma [62]. Thus, ECRG2 may function differently in certain cancer types depending on the tissue origin.

Multiple somatic mutations of *ECRG2* have been reported in various human malignancies such as lung, stomach, endometrium, skin, and colon cancer [3], which may adversely affect ECRG2 structure or function. Studies by Lucchesi et al. [3] recently demonstrated that while the wild-type form of ECRG2 exhibited strong growth suppression in cancer cells, the tumor-derived ECRG2 V30E mutant (identified in human lung cancer) failed to inhibit cancer cell growth. Also, unlike the wild-type ECRG2, the V30E mutant was not able to negatively modulate HuR and XIAP proteins [3]. The mutant version also did not activate caspases 3, 8, and 9 or cleave PARP [3]. Furthermore, cells expressing the mutant version were more resistant to cancer drug treatments [3]. These studies demonstrate that somatic mutations in *ECRG2* abolish its tumor-suppressive activity in human cancer.

Patel et al. [4] have recently identified a naturally occurring *ECRG2* promoter variant that may affect the regulation of *ECRG2* expression. This promoter variant was initially realized and cloned from the genomic DNA extracted from A549 human lung cancer cells [4]. In these cells, two alleles of *ECRG2* promoter variant were found: one named ECRG2-full (longer variant), while the other was called ECRG2-del (shorter variant), which was missing eight nucleotides (TAGAATTC) at position −217 to −209 when compared with the longer variant [4]. Interestingly, analyzing the database of single nucleotide polymorphisms (dbSNP), Patel et al. [4] found that a DNA sequence corresponding to the ECRG2-del variant existed in the dbSNP database, which has been identified as the alternate allele of genomic variant rs3214447 [63]. According to information curated from 1000 Genomes Project Phase-3, ~38.5% of the world’s population harbors an alternate allele of the rs3214447 variant (TAGAATTC deletion) in one or both copies of the *ECRG2* promoter [64]. In further investigations, they found that the basal transcriptional activity of the ECRG2-full promoter (ref allele) was much higher than that of the shorter (alt) allele (~2:1 ratio) [4]. Similar observations have been reported in the Genotype-Tissue Expression (GTEx v8) database, where the alt allele of rs3214447 is correlated with lower *ECRG2/SPINK7* expression in normal esophageal mucosa [65]. Importantly, DNA damage-induced *ECRG2* promoter activation was also significantly higher with the full-length variant (ref allele) than with the short variant (alt allele). Further, the shorter variant (alt allele) was also defective in p53-modulated *ECRG2* promoter induction [4]. These in vitro studies indicate that TAGAATTC deletion within the *ECRG2* promoter appears to negatively impact basal levels of *ECRG2* promoter activity, p53-mediated transcriptional regulation, and *ECRG2* promoter activation by DNA damage in general. Given that the rs3214447 alt allele is found in about 38.5% of the world’s population and ECRG2 plays important roles in DNA damage response, it would be interesting to determine how *ECRG2* expression is regulated in the human population with the rs3214447 alt allele under DNA damage-induced reaction in cells.

## 6. Therapeutic Implications

Studies have demonstrated that the plasmid or virus-mediated expression of ECRG2 exhibited strong growth inhibition in cultured cancer cells [3,4], and adenovirus-delivered ECRG2 through intra-tumoral administration also showed significant suppression in tumors grown in animals [2]. Further, the loss of *ECRG2* expression is frequently observed in multiple human malignancies [1,4,9]. Thus, restoring *ECRG2* expression and function in cancer cells is of great interest and an attractive therapeutic strategy. Several recent studies have investigated the effect of synthetic ECRG2 polypeptides on cultured cancer cells [66,67]. ECRG2 is a small protein of 85 amino acids; thus, its production is amenable to chemical synthesis. Studies by Song et al. [66,68] showed that synthetic ECRG2 alone (8.5 µg/L) killed esophageal cancer cells (EC9706) in cell cultures by ~18–25% at 24 and 72 h time intervals. The synthetic ECRG2 was also able to enhance cisplatin-induced cell death in cisplatin-resistant esophageal cancer cells [67]. These studies suggest that ECRG2 appears to have therapeutic potential for cancer treatment. However, while partial cell killing was observed in cells, the efficiency of uptake of the synthetic ECRG2 peptide into cells was not evaluated [66,67,68]. ECRG2 is a multifunctional protein that acts extracellularly to regulate cell membrane proteins such as the uPA-uPAR complex and also intracellularly to modulate proteins such as HuR and metallothionein 2A [1,3]. Thus, proper delivery of ECRG2 across the cell membrane is an important issue that needs to be considered. Further, ECRG2 is regulated by post-translational modification [3,4,69]. It is possible that protein modification (which is not present in synthetic peptides) may be required for the full function of ECRG2. For that reason, the synthetic ECRG2 peptide may not be very efficient in cell killing or performing other functions. Thus, newer approaches are necessary for the more efficient delivery of ECRG2 protein. In this context, several intracellular protein delivery systems have recently been evaluated for their efficiency in delivering proteins in animal studies and clinical trials. These include the cell-penetrating peptide (CPP)-based system [70], nanocarrier delivery system [71], and eTAT chimeric peptide delivery system [72]. With more efficient protein delivery approaches, it will be interesting to further evaluate the therapeutic potential of ECRG2 in future studies.

## 7. Conclusions

**Figure 2:**
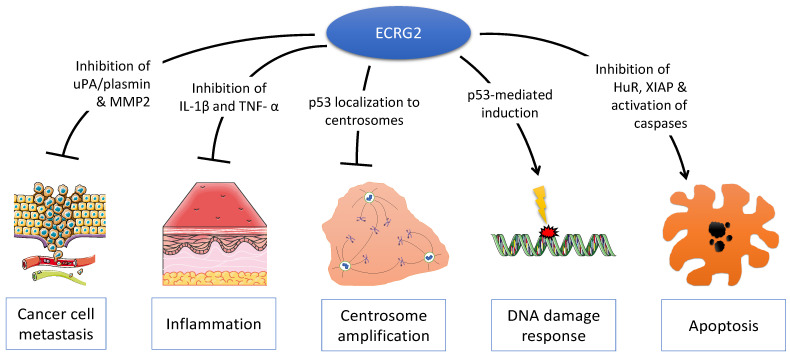
Multiple molecular functions of ECRG2. ECRG2 is a pleiotropic protein that plays important roles in diverse cellular phenotypes including cancer cell metastasis, inflammation, centrosome duplication, DNA damage response, and cell death by apoptosis.

Since its discovery, multiple lines of evidence have demonstrated that *ECRG2/SPINK7* is an important tumor suppressor, which is instrumental in numerous cellular phenotypes (Figure 2). *ECRG2/SPINK7* inhibits cancer cell migration and invasion and also suppresses tumor metastasis in animals [2,5,31]. The overexpression of *ECRG2/SPINK7* causes cancer cell death via multiple mechanisms [1,3,4]. Reduced or absent *ECRG2/SPINK* expression occurs in various human malignancies; cancer patients with low *ECRG2/SPINK7* expression in their tumor tissues exhibit shorter disease-free survival [1,4,9]. Importantly, *ECRG2/SPINK7* acts as an important player in DNA damage response and serves as a p53 transcriptional target for inducing cell death in DNA-damaged cells [4]. Thus, evidence indicates that defective ECRG2/SPINK7 (due to reduced expression or mutations) may be one of the important factors in human cancer development and acquisition of anticancer drug resistance. All lines of evidence signify the importance of *ECRG2/SPINK7* in human tumorigenesis and suggest that restoring *ECRG2/SPINK7* function in cancer cells may be an attractive therapeutic strategy. With a suitable protein delivery approach, *ECRG2* protein may prove to be a valuable anticancer therapeutic strategy.
